# Micelle-Mediated PbBr_2_ Complexation: Influence of Block Copolymer Architecture and Mixing Conditions

**DOI:** 10.1021/acs.langmuir.5c04107

**Published:** 2025-12-03

**Authors:** Belda Amelia Junisu, Ya-Sen Sun

**Affiliations:** Department of Chemical Engineering, 34912 National Cheng Kung University, Tainan 701, Taiwan

## Abstract

Block copolymer micelles mediate PbBr_2_ complexation through two distinct pathways: an architecture-dependent interfacial process under static conditions and a shear-dominated mechanism under dynamic mixing. This study reveals how the polystyrene-*block*-poly­(2-vinylpyridine) (PS-*b*-P2VP) asymmetry governs the chain adsorption conformations (*trains*, *loops*, and *tails*) on PbBr_2_ surfaces and its complexation outcome. Under static conditions, the process is kinetically limited by micellar diffusion. Upon reaching the PbBr_2_ surface, the strong pyridine-Pb^2+^ coordination induces a restructuring of the micelle core. For asymmetric PS-*b*-P2VP with long P2VP blocks, a thin PS shell allows the P2VP core to deform and spread onto the surface, creating a large adsorption footprint. The long-tethered P2VP chains form multiple *train*–*loop* segments, maximizing the number of coordination sites per micelles and enabling efficient PbBr_2_ dissociation into [PbBr_3_]^−^ complexes. Conversely, the thick PS shells of symmetric copolymers act as a steric barrier. This shield prevents interfacial restructuring and hinders complexation. Dynamic mixing introduces an energy-driven mechanism that circumvents these architectural constraints. Shear forces simultaneously fragment PbBr_2_ monoliths into nanoparticles, accelerating complexation independently of copolymer architecture. UV–vis and dynamic light scattering (DLS) analyses show that micelle dimensions and chain conformations dictate the efficiency of PbBr_2_ complexation. By correlating micelle architecture with mixing conditions, this work establishes key design principles for PbBr_2_ complexation. We reveal a critical mechanistic switch. Under static conditions, the process is architecture-dependent. In contrast, dynamic mixing creates a shear-dominated, architecture-independent process. This finding provides fundamental insight into controlling lead halide precursor solutions.

## Graphical abstract

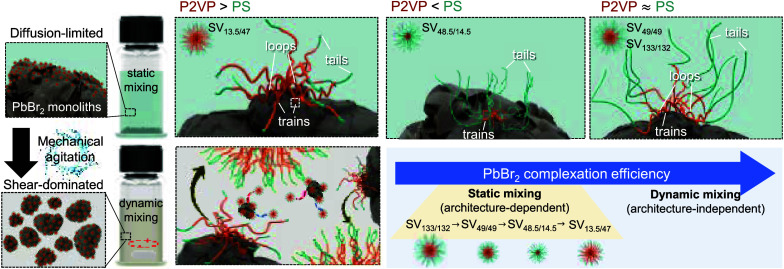

## Introduction

Templated growth of perovskite materials using self-assembling block copolymers has attracted considerable attention due to its potential for creating low-cost, high-efficiency optoelectronic devices.^1–6^ Polystyrene-*block*-poly­(2-vinylpyridine) block copolymers (PS-*b*-P2VP BCPs) have proven particularly effective as soft colloidal templates for synthesizing perovskite quantum dots.^7–14^ These amphiphilic block copolymers can self-assemble into a variety of nanostructures,^1,15,16^ facilitating the encapsulation and stabilization of inorganic nanoparticles.^17,18^ Our previous studies demonstrated a multistage synthesis process including PS-*b*-P2VP micellization, hierarchical emulsion formation, PbBr_2_ complexation, P2VP-complex coordination, and perovskite crystallization.^19–21^ 1,3,5-Trimethylbenzene (TMB) serves as a selective solvent, being favorable for the PS block but poor for both P2VP and PbBr_2_. Notably, PbBr_2_ cannot dissolve in TMB on its own. However, when PS-*b*-P2VP is introduced to facilitate hierarchical emulsification under stirring, PbBr_2_ monoliths can be effectively dispersed in TMB.

Occurring in various stages, PbBr_2_ complexation and P2VP-complex coordination within micellar cores are critical for producing encapsulated perovskite quantum dots.^8^ In previous reports, lead halide complexation was documented with an emphasis on the function of primary amine ligands.^22–24^ The underlying roles of block copolymers, such as PS-*b*-P2VP, were not fully recognized. This study aims to gain an in-depth understanding of the correlations between PbBr_2_ complexation and P2VP-complex coordination via static or dynamic mixing. Specifically, PbBr_2_ complexation involves the surface adsorption of PS-*b*-P2VP micelles from a selective solvent (TMB, used in this study) onto PbBr_2_ monoliths. Consequently, PbBr_2_ complexation and polymer–complex coordination occur at the interface between adsorbed P2VP chains and PbBr_2_ monoliths. The kinetics and dynamics of these interactions are influenced by the conformations of the adsorbed PS-*b*-P2VP chains and the surface adsorption kinetics of PS-*b*-P2VP micelles. These effects can be finely tuned by varying the molecular weights of PS-*b*-P2VP.

### Materials, Methods, and Instruments

Four polystyrene-*block*-poly­(2-vinylpyridine) block copolymers (PS-*b*-P2VP BCPs), PS13.5-*b*-P2VP47 (*M*_*n*,PS_ = 13.5 kg/mol, *M*_*n*,P2VP_ = 47 kg/mol, *M_w_*/*M_n_* = 1.11), PS48.5-*b*-P2VP14.5 (*M_n_*_,PS_ = 48.5 kg/mol, *M_n_*_,P2VP_ = 14.5 kg/mol, *M_w_*/*M_n_* = 1.07), PS49-*b*-P2VP49 (*M*_*n*,PS_ = 49 kg/mol, *M_n_*_,P2VP_ = 49 kg/mol, *M_w_*/*M_n_* = 1.08), and PS133-*b*-P2VP132 (*M_n_*_,PS_ = 133 kg/mol, *M_n_*_,P2VP_ = 132 kg/mol, *M_w_*/*M_n_* = 1.15) were purchased from Polymer Source, Inc. and used as received. For brevity, the polymers will be termed SV_*n*/*m*_, where “*n*” and “*m*” are the molecular weights of PS (S) and P2VP (V) blocks, respectively. PbBr_2_ was obtained from Sigma-Aldrich. 1,3,5-Trimethylbenzene (TMB, C_6_H_3_(CH_3_)_3_) was purchased from Thermo Scientific.

PS-*b*-P2VP BCPs were dissolved in 1,3,5-trimethylbenzene (TMB) to prepare BCP solutions, with a concentration of 1 mg/mL PS-*b*-P2VP in TMB. Subsequently, 1 mg of PbBr_2_ powder was added to each BCP solution to prepare two sets of precursor solutions. The first set of precursor solutions was left unstirred at room temperature for varied durations (0.5–40 h). For comparison, a second set of precursor solutions was stirred at a controlled speed (700 rpm) for the same durations to promote hierarchical emulsification. The resulting solutions were labeled ^sm^PRE_*x*,*n*/*m*_ and ^dm^PRE_*x*,*n*/*m*_ for the unstirred and stirred sets, respectively. The superscript “sm” and “dm” denote the static mixing and dynamic mixing processes, subscript “*x*” denotes the duration of storage (or stirring) in hours, and “*n*/*m*” represents the specific molecular weight of PS and P2VP copolymer, respectively.

UV–vis absorbance spectra were acquired by using a JASCO V-770 UV–vis/near-IR spectrophotometer to monitor the formation of lead bromide complexes as a function of time. Dynamic light scattering (DLS) measurements (Malvern Instruments Zetasizer Nano ZS) were used to determine the structural dimensions of the micelles in undiluted solutions. Simultaneous small-angle and wide-angle X-ray scattering (SAXS/WAXD, BL 13A NSRRC Taiwan) measurements were performed to probe the temporal evolution of the dispersed PbBr_2_/micelle in the stirred precursor solutions (^dm^PRE_*x*,*n*/*m*_). Surface morphologies of the dried ^sm^PRE_*x*,*n*/*m*_ and ^dm^PRE_*x*,*n*/*m*_ solutions (*x* = 6 and 40) were characterized using an atomic force microscope (AFM100Plus, Hitachi) in the tapping mode and using a transmission electron microscope (TEM, Hitachi H-7500) used at 80 kV.

## Results and Discussion

### Static Mixing

Figure 1 presents the intensity-weighted DLS profiles and UV–vis absorption spectra of PS-*b*-P2VP/PbBr_2_ precursor solutions, prepared with varying PS-*b*-P2VP molecular weights and storage durations (0–40 h). Four PS-*b*-P2VP copolymers were investigated: SV_13.5/47_ (short PS, long P2VP), SV_48.5/14.5_ (long PS, short P2VP), and SV_49/49_ and SV_133/132_ (two with symmetrical block lengths). DLS of neat PS-*b*-P2VP solutions (black curves in Figure 1a–d) reveals single and narrow peaks, indicative of well-defined monodisperse micelles. Intensity-weighted hydrodynamic diameters (*d*_H,int_) were 56, 52, 71, and 296 nm for the SV_13.5/47_ (^sm^PRE_0,13.5/47_), SV_48.5/14.5_ (^sm^PRE_0,48.5/14.5_), SV_49/49_ (^sm^PRE_0,49/49_), and SV_133/132_ (^sm^PRE_0,133/132_) systems, respectively. The neat micelles were also characterized using TEM and SAXS. The slight discrepancy between the micelle dimension from DLS and SAXS is discussed further in the Supporting Information (Figure S1 and Table S1).

**Figure 1:**
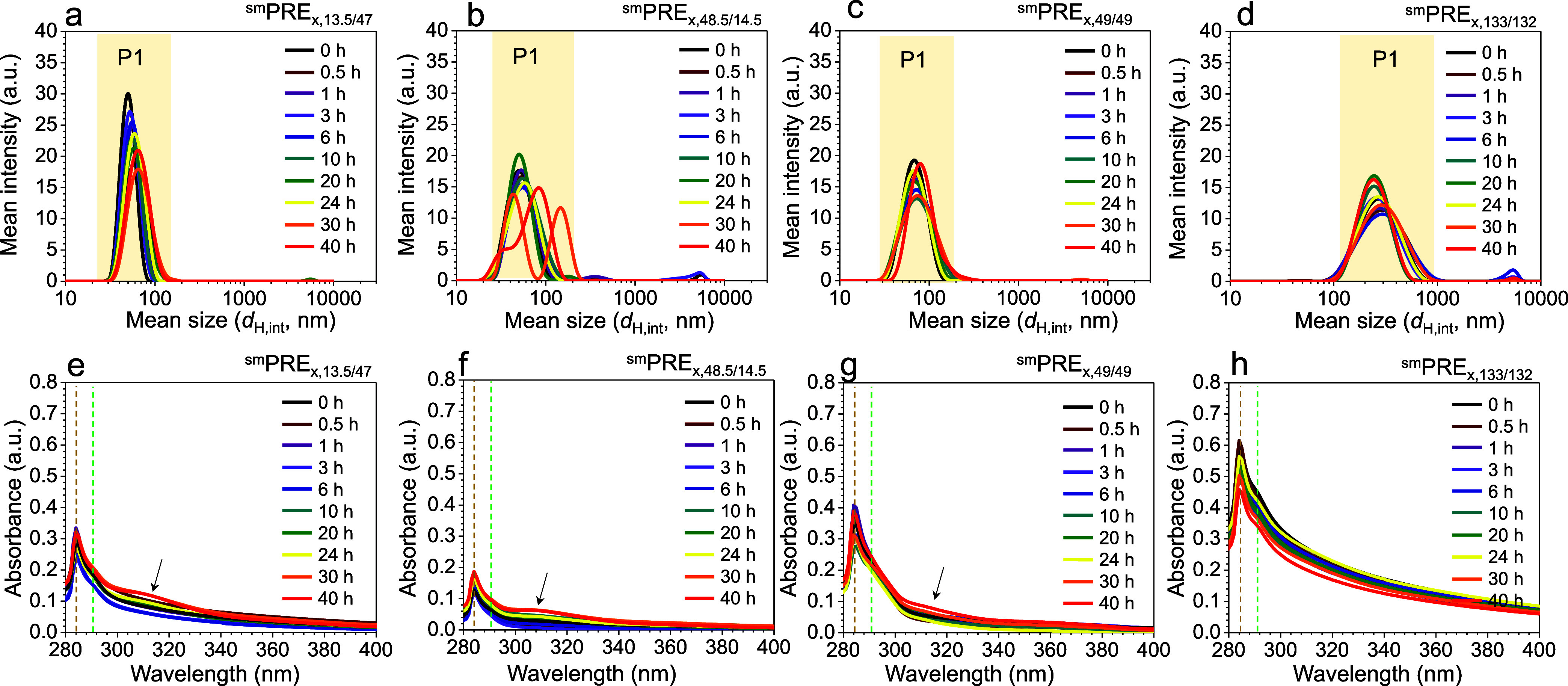
(a–d) Intensity-weighted DLS profiles and (e–h) UV–vis absorption spectra of the ^sm^PRE_*x*,*n*/*m*_ precursor solutions as a function of time (*x*: 0–40 h) and copolymer molecular weight. Data are shown for (a, e) ^sm^PRE_*x*,13.5/47_, (b, f) ^sm^PRE_*x*,48.5/14.5_, (c, g) ^sm^PRE_*x*,49/49_, and (d, h) ^sm^PRE_*x*,133/132_. The yellow highlighted regions in (a–d) correspond to Peak 1 (P1). Brown and green dashed vertical lines in (e–h) indicate persistent absorbance bands of solutions in all copolymer systems in TMB.

Following the addition of PbBr_2_, the asymmetric PS-*b*-P2VP systems (^sm^PRE_*x*,13.5/47_ and ^sm^PRE_*x*,48.5/14.5_) exhibited slow evolution under static conditions, as reflected by gradual increases in *d*_H,int_ (Figure 1a,b). For ^sm^PRE_*x*,48.5/14.5_, a pronounced bimodal distribution emerges after 30 h (Figure 1b). It is important to note that Figure 1a–d presents intensity-weighted size distributions, which are intrinsically biased toward larger structures, since the scattering intensity scales with the sixth power of particle radius (*I* ∝ *r* ^6^).^25^ Therefore, the apparent bimodality should not be interpreted as the dominance of large aggregates. Rather, even a very small amount of larger structures can generate a strong peak in the intensity-weighted DLS distribution due to the *r* ^6^ dependence of scattering intensity. To verify this interpretation, the number-weighted DLS spectra were analyzed for the ^sm^PRE_*x*,*n*/*m*_ solutions (Figure S2 in the Supporting Information). These results confirm that in both ^sm^PRE_*x*,13.5/47_ and ^sm^PRE_*x*,48.5/14.5_ systems, the smaller primary micelles remain the predominant structures by number throughout the 40 h period. Furthermore, the number-weighted hydrodynamic diameters (*d*_H,num_) in these two systems show a gradual increase over time (Figure S3a).

Under static (unstirred) conditions, the transport of micelles to the PbBr_2_ surface is governed by diffusion. The slow kinetics observed over 40 h are consistent with a process that is influenced not only by diffusion but also by the energetic adsorption at the particle surface. This behavior, where the overall rate is governed by a combination of mass transport and a potential activation barrier for surface attachment and reorganization, is a well-established phenomenon in the kinetics of polymer and colloid adsorption.^26,27^

In contrast, the ^sm^PRE_*x*,49/49_ and ^sm^PRE_*x*,133/132_ solutions using SV_49/49_ and SV_133/132_ showed negligible changes in *d*_H_ and scattering intensity over time (Figures 1c,d and S2c,d). The stark difference in kinetics between the symmetric and asymmetric systems can be attributed to the role of the PS shell as a diffusion barrier for the PbBr_2_ complex. For SV_49/49_ and SV_133/132_, the long PS blocks form a densely packed shell around the P2VP core. The mobility of these entangled shell chains is restricted, creating a significant kinetic barrier that hinders the transport of PbBr_2_ into the core, where the reactive P2VP sites reside. Consequently, complexation is kinetically arrested, and the DLS profiles remain unchanged. This is confirmed by the consistent dominance of Peak 1 (P1) in DLS profiles (Figures 1c,d and S2c,d), which is a marker of micelles retaining hydrodynamic diameters comparable to the neat solutions. Visually, the nonstirred precursor solutions (^sm^PRE_*x*,*n*/*m*_) for the symmetric BCPs remained transparent over 40 h (Figure S4, upper panel), consistent with minimal macroscopic changes. While minor visual variations may arise from lighting conditions, the quantitative number-weighted DLS data (Figure S3a) confirmed little to no change in hydrodynamic diameter, and the PDI values remained below 0.4 (Figure S3b), further supporting the absence of turbidity. In contrast, the asymmetric SV_13.5/47_ copolymer, with its significantly shorter PS block, possesses a much thinner shell, reducing the diffusion barrier for PbBr_2_ penetration and enabling the observed complexation-driven micelle growth in the ^sm^PRE_*x*,*n*/*m*_ solutions.

To provide further insight into PbBr_2_ complexation within the BCP colloidal solution, UV–vis absorption spectra were collected. Both absorption and scattering are observed in the spectra, with scattering being particularly pronounced for the ^sm^PRE_*x*,133/132_ solutions (Figure 1h). It is important to note that absorption and scattering differ fundamentally in their origins and effects.^28^ Mie scattering (for particle diameters comparable to or larger than the wavelength, *d* ∼ λ or *d* ≫ λ) and Rayleigh scattering (for *d* ≪ λ) are inevitable when colloidal solutions are characterized via absorption spectroscopy.

As shown in Figure 1e–h, sharp peaks at ∼285 nm with shoulders centered at ∼291 nm were observed. While isolated phenyl rings absorb at 260 nm,^29^ the observed shift to ∼285 nm should be attributed to interphenyl interactions of PS in TMB. This assignment is confirmed by the spectrum of pure PS in TMB, which shows a similar peak at ∼286 nm (Figure S5). Additionally, the shoulder at ∼291 nm likely originates from the absorbance of micelle-dispersed PbBr_2_ particles.^30,31^ Both features appear consistently, regardless of the molecular weight of PS-*b*-P2VP (dashed lines in Figure 1e–h). After about 20 h, a new absorption shoulder emerges between ∼300 and 330 nm. This shoulder is prominent for asymmetric PS-*b*-P2VP copolymers, whereas symmetric PS-*b*-P2VP copolymers display relatively weaker intensity in the same region. The trend aligns with the DLS results, where asymmetric PS-*b*-P2VP copolymers showed a more pronounced increase in size. These findings suggest that the broad shoulder centered at ∼310 nm peak may indicate the formation of P2VP-capped complexes. The absorbance intensity of this band reflects the extent of complexation, which is greater in asymmetric systems.

The kinetics of PbBr_2_ complexation are critically dependent on the block copolymer architecture, with asymmetric designs showing markedly higher efficiency than their symmetric counterparts. The ^sm^PRE_*x*,13.5/47_ solutions using SV_13.5/47_, featuring a short PS shell block and a long P2VP core block, demonstrate the most rapid complexation. Its thin PS shell presents a minimal barrier to ion transport, allowing PbBr_2_ to readily access the P2VP core. The result in Figure 1a,e is substantial, showing uniform micellar growth while maintaining a spherical morphology and narrow size distribution. The retention of monodisperse, spherical micelles after static stirring is confirmed by TEM characterization (Figure S6).

In stark contrast, copolymers with relatively thick PS shells exhibit significantly hindered complexation dynamics (Figure 1c,d,g,h). For the ^sm^PRE_*x*,49/49_ and ^sm^PRE_*x*,133/132_ solutions using SV_49/49_ and SV_133/132_, this inefficiency is 2-fold. First, the thick PS shell acts as a physical barrier, impeding both the diffusion of ions into the P2VP core and the conformational rearrangements of the core chains required for binding. Second, these architectures form large micelles (*d*_H,int_ up to 296 nm), which drastically slow their translational diffusion. Based on the Stokes–Einstein equation,^32^ the diffusion coefficient (*D*) for the largest symmetric micelle (*D* ≈ 2.3 × 10^–12^ m^2^/s) is an order of magnitude smaller than that of a smaller asymmetric micelle (*D* ≈ 1.3 × 10^–11^ m^2^/s), severely limiting the rate at which they encounter PbBr_2_ surfaces in an unstirred solution. Based on the Einstein–Smoluchowski relation,^33^ the smaller SV_48.5/14.5_ micelle would take approximately 0.042 s, while the larger SV_133/132_ micelle would require nearly 0.220 s (see Table S2). The largest symmetric micelle (SV_133/132_) requires more than 5 times longer to travel compared to the smallest asymmetric micelle (SV_48.5/14.5_), providing a quantitative basis for the observed differences in complexation kinetics under diffusion-controlled conditions. Collectively, these findings reveal that the BCP shell thickness and the resulting micelle size are the dominant architectural parameters governing the complexation pathways.

### Dynamic Mixing

Stirring enhances PbBr_2_ complexation in PS-*b*-P2VP/TMB colloidal solutions through two primary mechanisms. First, stirring facilitates the rapid breakdown of PbBr_2_ monoliths into smaller particles. These smaller particles disperse more readily in the TMB, increasing their available surface area for colloidal adsorption. Second, stirring promotes dynamic collisions between PS-*b*-P2VP micelles and PbBr_2_ fragments, accelerating coordination at P2VP core sites. To investigate these effects, precursor solutions were stirred continuously at 700 rpm from 0 to 40 h, and their evolution was monitored via DLS and UV–vis spectroscopy. Figure 2 presents the DLS size distributions and UV–vis absorption spectra of the stirred precursor solutions (^dm^PRE_*x*,*n*/*m*_) under dynamic mixing. A gradual color change of the stirred precursor solution from transparent bluish to milky white indicates the formation of a dispersion (see bottom panel in Figure S4).

**Figure 2:**
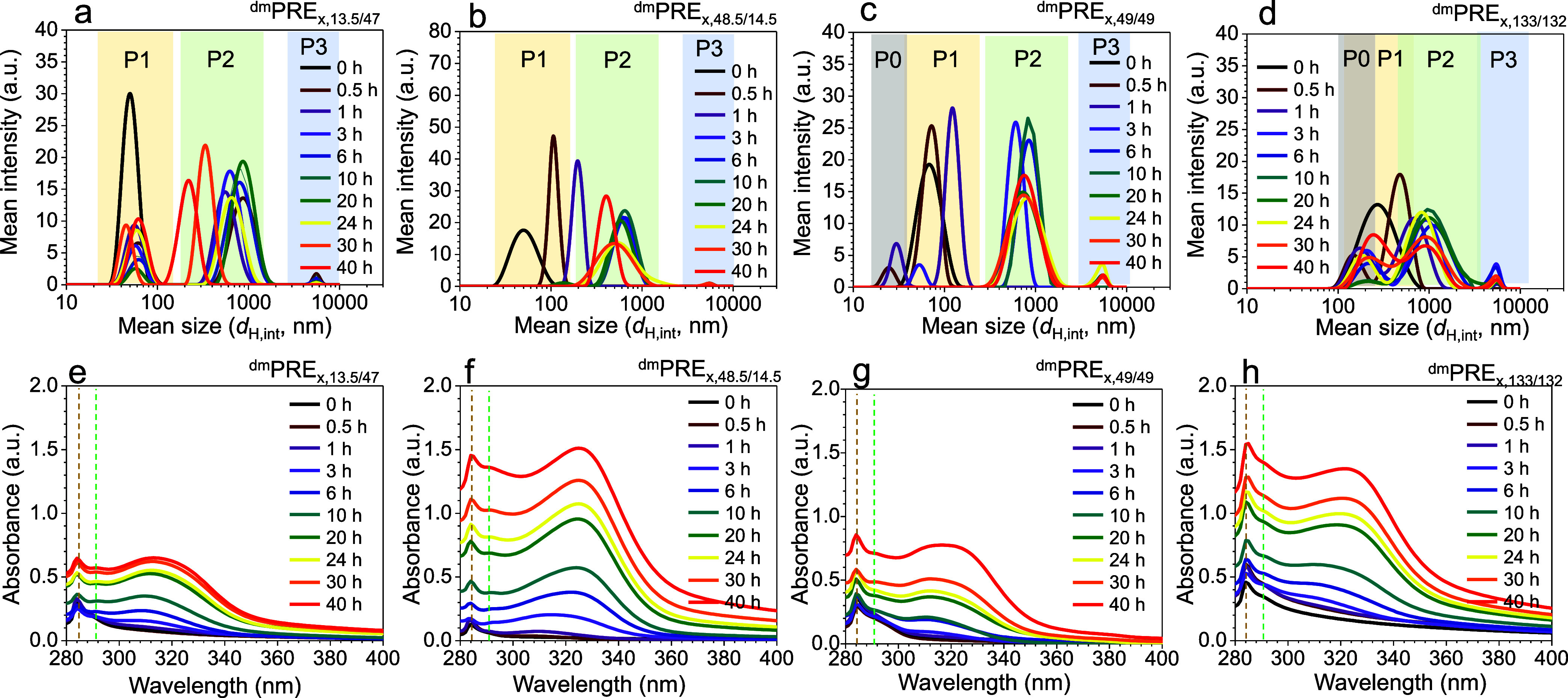
(a–d) Intensity-weighted DLS profiles and (e–h) UV–vis absorption spectra of ^dm^PRE_*x*,*n*/*m*_ precursor solutions as a function of stirring time (*x*: 0–40 h) and the molecular weight of the copolymer. Data are shown for (a, e) ^dm^PRE_*x*,13.5/47_, (b, f) ^dm^PRE_*x*,48.5/14.5_, (c, g) ^dm^PRE_*x*,49/49_, and (d, h) ^dm^PRE_*x*,133/132_. Yellow, green, and blue highlighted areas in (a–d) correspond to Peak 1 (P1), Peak 2 (P2), and Peak 3 (P3), respectively. Gray highlighted areas in (c, d) denote Peak 0 (P0). Brown and green dashed vertical lines in the UV–vis spectra indicate the characteristic absorbance band of PS-*b*-P2VP in TMB.

Stirring has dynamic effects on the size and distribution of the dispersed nanoparticles. Unlike static mixing, dynamic mixing generates polydisperse populations, as evidenced by multiple DLS peaks (Figures 2a–d and S7). These peaks likely correspond to distinct species: primary micelles (P1), fragmented micelles (P0), micelles-fused PbBr_2_ fragments (P2), and dispersed PbBr_2_ nanoparticles (P3). Peaks are labeled relative to the size of primary micelles in the neat solution (*x* = 0 h), with P0 representing objects smaller than the neat micelles and P2 and P3 representing larger objects. TEM characterization of the dried precursor solutions (^dm^PRE_*x*,*n*/*m*_) visually confirms the presence of these multiple populations (Figure S8). Note that the P3 species is not shown due to its exceptionally large dimensions.

Shifts in *d*_H_ reflect transient adsorption/desorption equilibria at PbB_2_ interfaces, mediated by PS-*b*-P2VP micelles that dynamically stabilize hierarchical emulsions. The temporal evolution of PbBr_2_-micelle dispersions under stirring was characterized by simultaneous ex situ SAXS and WAXD (Figure S9). For all copolymers, prolonged stirring progressively enhanced contributions from the secondary structures. Upon stirring, PbBr_2_ microparticles become well-dispersed in TMB through a colloid-assisted microemulsion process. These microparticles primarily consist of orthorhombic PbBr_2_ crystals (PDF#031-0679). Unlike PbBr_2_ complexation and phase transformations of crystals observed in the PS-*b*-PEO system,^34^ the presence of PS-*b*-P2VP in this study results only in PbBr_2_ complexation.

Quantitative analysis of Figure 2a–d highlights the sequential emergence of Peaks P0 and P3 across all PS-*b*-P2VP systems (see Figure S10). The early appearance of P2 under dynamic mixing conditions (observed even at initial time points) suggests rapid micelle adsorption and fusion with dispersed PbBr_2_ fragments. Notably, only SV_49/49_ and SV_133/132_ precursor solutions exhibit a distinct P0 fraction during early stirring, likely due to shear-induced fragmentation of primary micelles.

While the interpretation of DLS in stirred systems is inherently more complex, the present results provide a key mechanistic insight. Mechanical agitation does not simply accelerate complexation; it changes the dominant kinetic regime. Under static conditions, complexation is governed by molecular-weight-dependent micellar diffusion and architecture-driven transport through the PS shell. In contrast, stirring shifts the system into a shear-dominated regime, where shear-assisted fragmentation of PbBr_2_ and convective transport prevail. These processes overcome the steric and kinetic barriers that previously limited the complexation in the symmetric copolymers, enabling their effective participation in complex formation.

Figure 2e–h shows the time-dependent UV–vis absorption spectra of the ^dm^PRE_*x*,*n*/*m*_ solutions under dynamic mixing. Three key trends are evident across all systems. First, absorbance bands at ∼285 and ∼291 nm persist across all molecular weights of PS-*b*-P2VP (dashed vertical lines in Figure 2e–h). These absorbance bands correspond to electronic transitions of the PS and micelle-dispersed PbBr_2_ particles in TMB. Second, a progressive increase in spectral intensity over time, particularly within the 300–350 nm range, reflects ongoing formation of lead bromide complexes. Third, the asymmetric broadening of this feature suggests the coexistence of multiple coordination species, which is expected given the dynamic nature of the system.

Lead bromide complexes are well-known to exhibit characteristic UV–vis absorbance features that depend sensitively on the solvent environment, coordinating ligands, and polynuclear states.^30,31,35–39^ In particular, the mononuclear [PbBr_3_]^−^ complex has been reported to exhibit an absorbance maximum at wavelengths ranging from 306 to 320 nm.^30,31,34–36^ Because broad absorbance envelopes in this region can arise from multiple types of complexes, deconvolution is required to distinguish their individual contributions.^36^

In our system, all of the lead bromide complexes are sequestered within the P2VP micelle cores. These highly charged ionic species are insoluble in the nonpolar TMB solvent; therefore, their presence in solution directly indicates stabilization by the pyridine-rich P2VP environment. At elevated local concentrations of [PbBr_3_]^−^ complexes, some complexes preferentially interact with one another, forming higher-order polynuclear species that exhibit progressively red-shifted absorption bands.^36–39^ This coordination-density dependence accounts for the multiple spectral features observed. Within this physical framework, the UV–vis spectra reflect a distribution of lead bromide complex geometries, all stabilized to varying degrees by the P2VP cores.

In our analysis, the peak with a maximum between 307 and 310 nm is assigned to the mononuclear [PbBr_3_]^−^ complex, which represents the initial dissociation product of PbBr_2_ within the P2VP core. This assignment has been reported previously in the literatures.^30,31,35,36^ This correlation implies that increasing [PbBr_3_]^−^ concentrations directly reflect increased pyridine-mediated complexation. Other features, such as the broad absorption between 325 and 360 nm, likely represent a distribution of polynuclear lead bromide complexes (including [Pb_4_Br_11_]^−^, [Pb_2_Br_5_]^−^, and [PbBr_4_]^2–^ near 325, 345, and 357 nm, respectively).^30,31,35–39^ Additionally, broad absorption in the 370–380 nm range is attributed to PLB complexes with coordination states below [PbBr_4_]^2–^.^37^ To semiquantitatively track the evolution of these species, the UV–vis spectra of solutions with *x* = 0.5 h and *x* = 40 h in Figure 2e–h were isolated by Gaussian deconvolution based on these literature-validated assignments.^30,31,35–39^

The ^dm^PRE_*x*,*n*/*m*_ solutions comprise three primary components contributing to the overall absorbance: PS-*b*-P2VP micelles, lead bromide complexes, and dispersed PbBr_2_ nanoparticles. Centrifugation removes residual PbBr_2_ nanoparticles and partially eliminates unbound lead bromide complexes (Figure S11), significantly reducing scattering contributions. Therefore, to isolate signals specific to coordination chemistry, all UV–vis spectra were baseline-corrected to account for background contributions and scattering effects prior to analysis.^39^ Gaussian deconvolution was then applied to resolve seven distinct bands, each assigned to specific interactions or complexes with the maxima range of (i) ∼282 to 284 nm, (ii) ∼289 to 292 nm, (iii) ∼307 to 310 nm, (iv) ∼320 to 325 nm, (v) ∼343 to 345 nm, (vi) ∼357 to 360 nm, and (vii) ∼370 to 380 nm. Figure 3 quantifies the integrated areas of these deconvoluted peaks for all four PS-*b*-P2VP systems at 0.5 and 40 h of stirring. Note that the detailed assignment of each complex type may be different in different solvents other than TMB. Therefore, broad spectral ranges were used for fitting to accommodate solvent-dependent peak shifts, ensuring consistent cross-system comparisons.

**Figure 3:**
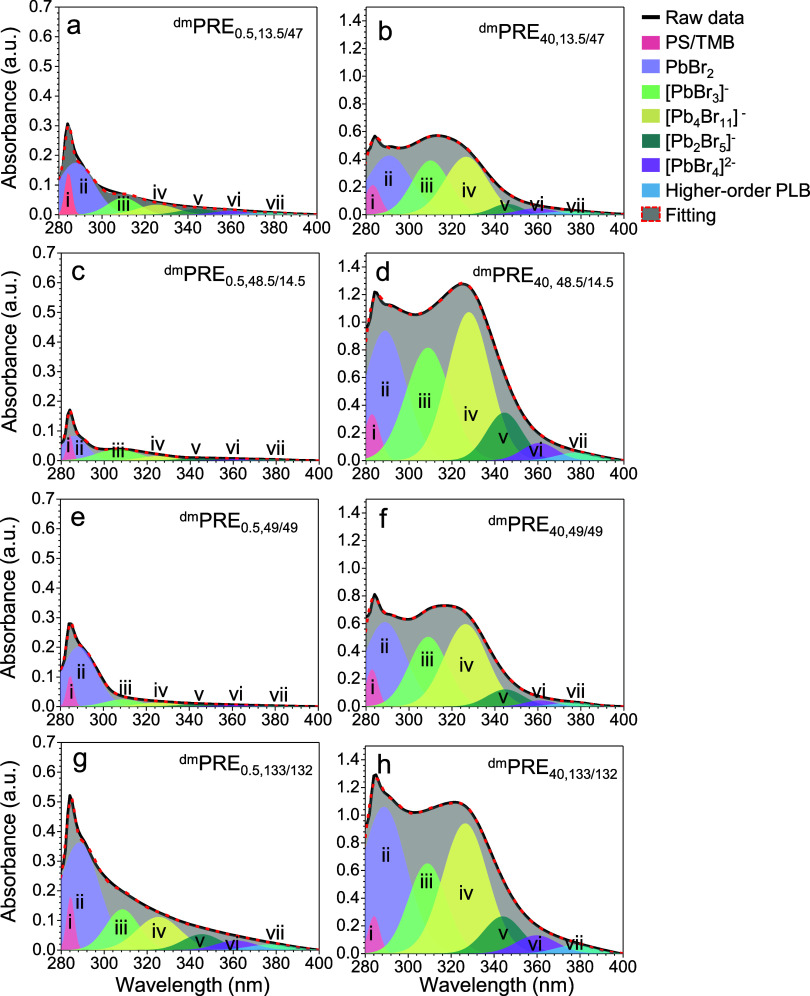
Experimentally measured UV–vis absorbance spectra of precursor solutions using different PS-*b*-P2VP copolymers. Panels (a–h) correspond to ^dm^PRE_0.5,*n/m*_ (left column) and ^dm^PRE_40,*n/m*_ (right column) solutions with *n/m* = (a,b) 13.5/47, (c,d) 48.5/14.5, (e.f) 49/49 and (g,h) 133/132. Gaussian deconvolution reveals seven distinct species-associated peaks, resolving contributions from (i) PS/TMB interactions (282–284 nm), (ii) micelle-dispersed PbBr_2_ particles (289–292 nm), (iii) [PbBr_3_]^−^ (307–310 nm), (iv) [Pb_4_Br_11_]^−^ (320–325 nm), (v) [Pb_2_Br_5_]^−^ (343–345 nm), (vi) [PbBr_4_]^2–^ (357–360 nm), and (vii) higher-order polynuclear lead bromide (PLB) complexes (370–380 nm). The superscript “dm” denotes dynamic mixing.

As Figure 3 shows, Gaussian deconvolution provides an excellent fit for the UV–vis absorbance spectra across all ^dm^PRE_*x*,*n*/*m*_ solutions. The intensities of Peaks iii–vii increase markedly over the 40 h stirring period, confirming the progressive accumulation of various lead bromide complex species. Compared to the static systems (Figure S12), which are governed by slow diffusion across a thick boundary layer, these stirred solutions exhibit significantly enhanced complexation kinetics. This acceleration is consistent with the established principle that mechanical agitation overcomes mass transfer limitations in heterogeneous systems, thereby promoting interactions between the micelles and particle surfaces.^40^ To quantify the temporal evolution of these complex populations, the integrated area and amplitude of each Gaussian peak were calculated and are tracked over time in Figure 4.

**Figure 4:**
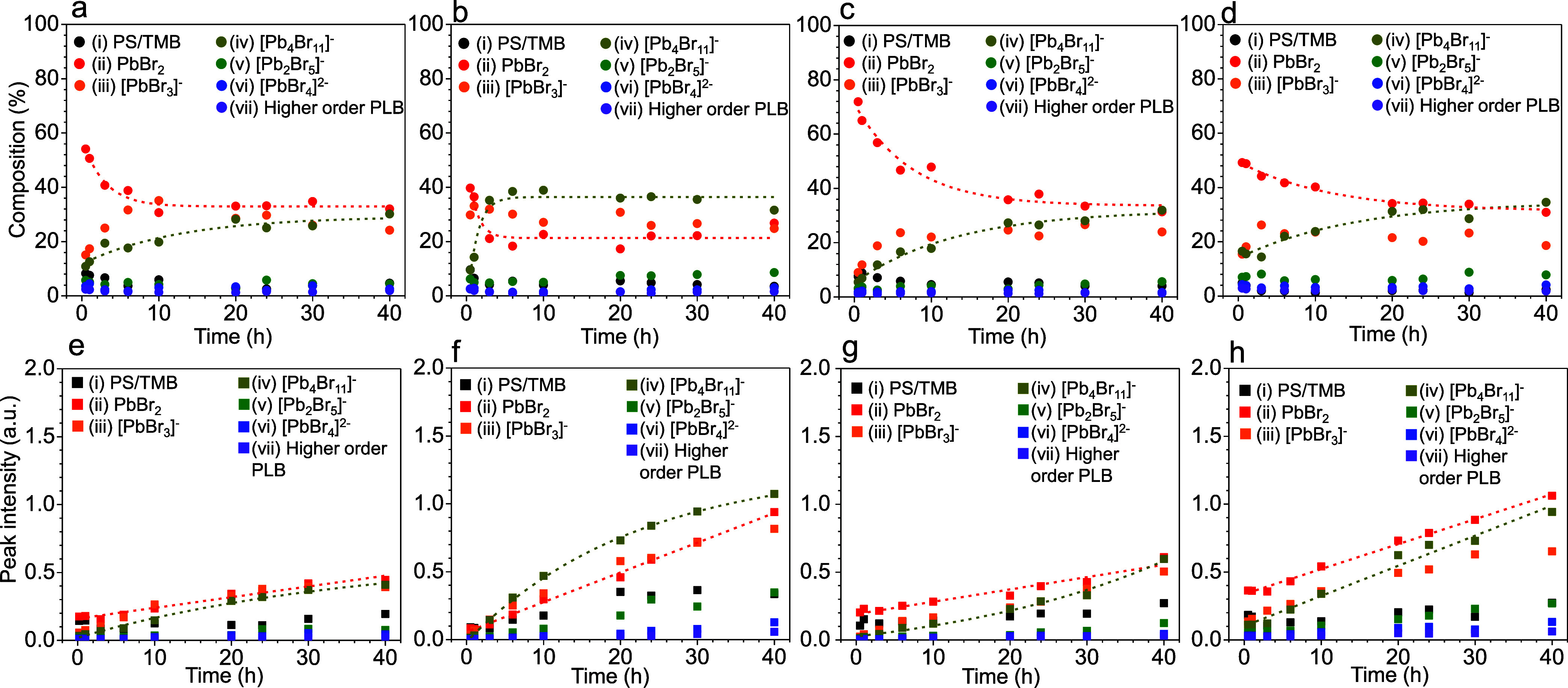
(a–d) Temporal evolution of lead bromide complex populations and (e–h) Gaussian peak intensities derived from UV–vis spectral deconvolution of stirred precursor solutions. Data are shown for (a, e) ^dm^PRE_*x*,13.5/47_, (b, f) ^dm^PRE_*x*,48.5/14.5_, (c, g) ^dm^PRE_*x*,49/49_, and (d, h) ^dm^PRE_*x*,133/132_. Red and lime dashed lines highlight trends for Peak ii (micelle-dispersed PbBr_2_, 289–292 nm) and Peak iv ([Pb_4_Br_11_]^−^ complexes, ∼320 to 325 nm), respectively.

Figure 4 reveals three critical insights into the PbBr_2_ complexation dynamics. First, Peak i (282–284 nm, PS/TMB interactions) remains stable over time, while Peak ii (287–291 nm, PbBr_2_ particles) decays exponentially (red dashed lines). This exponential decay coincides with a gradual rise in Peak iv (∼320 to 325 nm, polynuclear [Pb_4_Br_11_]^−^ complexes, lime dashed lines). The compensatory relationship suggests a dynamic equilibrium between PbBr_2_ particles and the [Pb_4_Br_11_]^−^ complexes. PbBr_2_ particles might dissolve and form a complex to form [Pb_4_Br_11_]^−^ complexes. This also explains the concurrent increase in Peak iii (307–310 nm, mononuclear [PbBr_3_]^−^) as more [PbBr_3_]^−^ complexes become stabilized by pyridine. Second, Peak v ([Pb_2_Br_5_]^−^, ∼343 to 345 nm), Peak vi ([PbBr_4_]^2–^, ∼357 to 360 nm), and Peak vii (higher-order PLB, 370–380 nm) show negligible temporal changes in both area and intensity (Figure 4e–h), indicating steady-state concentrations for these species. Finally, linear growth in the intensities of Peaks ii–iv implies continuous formation of early-stage complexes, despite compositional shifts among species. This kinetic hierarchy aligns with the mechanical-agitation-driven complexation pathway, where stirring prioritizes initial coordination over higher-order cluster assembly.

The micellization of PS-*b*-P2VP in TMB is driven by the differential solubility parameters (δ) of the solvent and polymer blocks. TMB (δ_TMB_ = 18 MPa^1/2^)^41^ selectively solvates the PS blocks over P2VP (δ_PS_ = 18.6 MPa^1/2^ and δ_P2VP_ = 19.8 MPa^1/2^),^42,43^ inducing strong core–shell micelle formation (Figure 5a_i_). Using other nonpolar solvents only results in weak micellization.^19^ Micelle dimensions, including core radius and shell thickness, are tunable via the copolymer’s molecular weight (Figure S1). The architecture of the PS-*b*-P2VP micelle in TMB directly modulates the accessibility of P2VP chains to PbBr_2_ surfaces. While the PS corona stabilizes micelles in the TMB, the P2VP cores retain dynamic flexibility to interact with PbBr_2_ microparticles. This interplay between micelle dimensions and polymer chain mobility governs the adsorption pathways critical to PbBr_2_ complexation kinetics.

**Figure 5:**
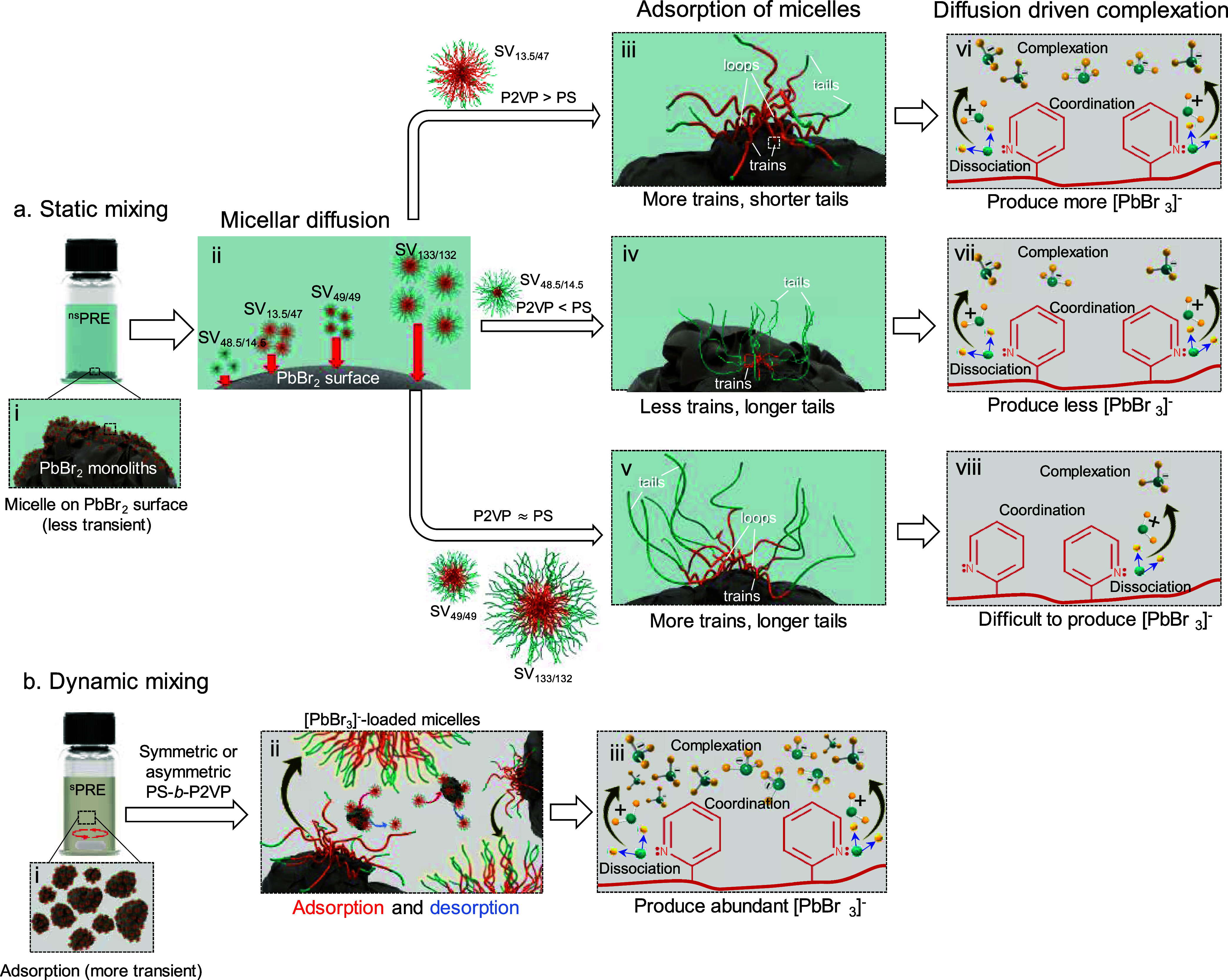
Schematic illustration of PbBr_2_ complexation in different precursor solutions: (a_i–viii_) static mixing (^sm^PRE_*x*,*n*/*m*_) and (b_i–iii_) dynamic mixing (^dm^PRE_*x*,*n*/*m*_). Arrows in image (a_ii_) indicate the estimated diffusion time of micelles onto the PbBr_2_ surface. Different conformations of polymer chains and complexation extent during static mixing were illustrated for (a_iii,vi_) SV_13.5/47_ with short PS and long P2VP, (a_iv,vii_) SV_48.5/14.5_ with long PS and short P2VP, and (a_v,viii_) SV_49/49_ and SV_133/132_ with symmetrical chain length.

The complexation of PbBr_2_ under static conditions is controlled by a sequence of interfacial phenomena. The process is initiated by the diffusion^44^ of individual PS-*b*-P2VP micelles through the TMB solvent to the PbBr_2_ particle surface (Figure 5a_ii_). The rate of diffusion is inversely proportional to the hydrodynamic diameter of the micelles (Table S2). Therefore, as established by our DLS data (see Figure 1), the larger symmetric copolymers are kinetically disadvantaged from the outset compared to their smaller, more mobile asymmetric counterparts.

Upon arrival at the PbBr_2_ surface, a micelle undergoes physical adsorption driven by favorable enthalpic interactions between the P2VP chains and PbBr_2_. The adsorption behavior of PS-*b*-P2VP has been investigated previously.^45,46^ Following adsorption, the chains adopt a specific interfacial conformation to balance the enthalpic gain of coordination against the entropic cost of immobilization.^47^ Scheutjens and co-workers theoretically demonstrated that, during the physical adsorption of a homopolymer from solution onto a flat solid substrate, the chain does not lie completely flat but instead adopts the classical *train*–*loop*–*tail* conformations.^48,49^ Only a fraction of the segments make direct contact with the surface, while the remainder extend outward into the surrounding solution. The surface-bound segments form *trains*, which are contiguous sequences of monomers lying flat on the substrate due to their favorable interactions with the surface. Between *trains*, segments may temporarily detach and return to the surface, forming *loops* whose two ends remain anchored. In addition, each polymer chain typically exhibits one or two *tails* originating from its free ends, which are anchored at only one end and extend into the solution. These *trains, loops, and tails* constitute the classical conformational picture of an adsorbed polymer chain, reflecting the balance between the enthalpic driving force for adsorption and the entropic cost of chain deformation. These conformations differ fundamentally from the brush- or mushroom-like conformations formed by chemically grafted polymers.^50^

The *train–loop–tail* framework has been further extended to describe the adsorption of diblock copolymers on flat surfaces.^51,52^ Importantly, the classical homopolymer adsorption model cannot fully capture the more complex behavior of adsorbed block copolymers, whose conformations are strongly influenced by solvent quality and the compositional fraction of each block.^53^ For instance, a nonadsorbing block tends to form *tails*, whereas an adsorbing block preferentially forms *trains* and *loops*.^51,52^

Directly determining the *loop* size or segment-level conformations within bound layers at the PbBr_2_–solution interface is exceptionally challenging. Techniques such as neutron reflectometry (NR)^54^ or small-angle neutron scattering (SANS)^55–58^ on selectively deuterated polymer chains could, in principle, provide depth-resolved density profiles that differentiate *train*-rich regions from *loop*/*tail*-dominated regions. However, such specialized measurements are beyond the scope of the present work, which focuses on the kinetic behavior and synthetic outcomes.

Moreover, we conducted additional SAXS, TEM, and AFM measurements (Figures S6, S8, and S9). While these characterizations help confirm morphological features, none of these techniques can resolve chain-level conformations such as *trains, loops, and tails*. These conformations occur at the subnanometer scale and require chemical contrast between adsorbed and nonadsorbed polymer segments. In our system, the chains are buried at a reactive solid–liquid interface, and the electron-density contrast between P2VP and PbBr_2_ is insufficient for direct imaging. TEM and AFM (Figures S6 and S8) cannot distinguish individual P2VP segments, and SAXS (Figure S9) provides only ensemble-averaged scattering profiles that do not resolve segment-specific adsorption, particularly in the presence of high-Z materials, such as PbBr_2_ macroparticles.

Nevertheless, the *train–loop–tail* framework has been widely used to develop a deeper understanding of bound layers formed by polymers adsorbed on flat substrates^54^ and on dispersed nanoparticles.^47,55–58^ In particular, current descriptions of bound layers rely largely on this classical model: polymer segments in direct contact with the nanoparticle surface form flattened *trains* as a dense region, while *loops* and extended *tails* give rise to a loose region.^47,55–57^ According to established theoretical and experimental studies,^47–59^ physically adsorbed PS-*b*-P2VP chains are expected to adopt the classic *train–loop–tail* conformations on PbBr_2_ surfaces. In this framework, *trains* represent P2VP segments directly coordinated to the surface through pyridine-Pb^2+^ lone-pair interactions, *loops* represent nonadsorbed P2VP segments that bridge adjacent *trains*, and *tails* consist of PS blocks solvated in TMB (Figure 5a_iii–v_). The relative prevalence of these conformations is governed by the block copolymer architecture and is consistent with the scenario described by Evers et al.^51,52^ Although these constrained conformations impose a significant entropic penalty, this penalty is compensated by two major enthalpic gains: favorable P2VP-PbBr_2_ coordination and favorable PS-TMB solvation.

Furthermore, unlike annealing-driven physical adsorption of BCP melts on non-neutral surfaces,^60^ the preferential adsorption of P2VP onto PbBr_2_ in our system is influenced not only by the strong affinity of pyridinic groups for Pb^2+^ cations and [Pb*_x_* Br_*y*_]^2*x*–*y*^ complexes but also by the solvent selectivity of TMB. TMB is a good solvent for PS but a nonsolvent for both P2VP and PbBr_2_. As a result, the PS block is preferentially solvated by TMB and is disfavored from adsorbing onto the PbBr_2_ surface. Consequently, when the PS block extends into solution as a dangling *tail*, it functions solely as a nonadsorbing steric layer that stabilizes the dispersion of PbBr_2_ particles rather than contributing to the enthalpic driving force for adsorption.

The specific architecture of each copolymer directly governs its interfacial conformation during the initial stages of complexation. For the asymmetric PS-*b*-P2VP copolymer (SV_13.5/47_), the thin PS shell and long P2VP chains facilitate the formation of a large adsorption footprint, enabling the creation of multiple *train* segments per chain and thus a high density of active coordination sites (Figure 5a_iii_). In contrast, architectures with thick PS shells face significant steric barriers that prevent this effective core restructuring. The asymmetric SV_48.5/14.5_ system is forced into a less efficient, *tail*-dominated conformation (Figure 5a_iv_), while the symmetric copolymers (SV_49/49_ and SV_133/132_) are limited to *loop*- and *tail*-dominated states with minimal surface contact (Figure 5a_v_).

Finally, the overall rate of the process is modulated by diffusion-driven complexation, which acts as a kinetic bottleneck, determining how frequently these adsorption events can occur. As established in our previous works,^19–21^ the formation of hierarchical emulsions in PS-*b*-P2VP/PbBr_2_ colloidal systems proceeds through distinct micro- and nanoscale processes. The chemical reaction itself is initiated exclusively by the *train* segments. Their direct pyridine-Pb^2+^ coordination induces the dissociation of PbBr_2_ into Pb^2+^ and Br^–^, leading to the formation of [PbBr_3_]^−^ complexes stabilized within the P2VP core (as illustrated in Figure 5a_vi_). The *train*-rich SV_13.5/47_ system is therefore the most efficient at generating stable complexes. Although the asymmetric SV_48.5/14.5_ system follows the same pathway, its *tail*-dominated conformation leads to less efficient dissociation (Figure 5a_vii_). The symmetric copolymers (SV_49/49_ and SV_133/132_), lacking sufficient *train* segments and further limited by slower diffusion, exhibit a dramatically diminished capacity for PbBr_2_ dissociation. Consequently, their overall complexation efficiency is the lowest (Figure 5a_viii_).

Under dynamic mixing, the complexation mechanism transitions from a diffusion-controlled to a shear-dominated process. The continuous application of shear serves three primary functions that overcome the limitations observed in static systems. First, it facilitates the mechanical fragmentation of PbBr_2_ monoliths into nanoparticles (Figure 5b_i_), dramatically increasing the total available surface area for reaction. Second, shear replaces slow micellar diffusion with rapid convection, ensuring a high flux of micelles to the particle surfaces. This process bypasses the kinetic bottleneck faced by large, slow-moving micelles such as SV_49/49_ and SV_133/132_. Finally, the high-energy collisions in the shear field induce a state of transient adsorption and desorption at the particle interface (Figure 5b_ii_). These nonequilibrium conformations continuously disrupt the ordered conformations seen in static systems and physically overcome the steric barrier of thick PS shells. Thus, this new regime leads to the complex, polydisperse populations observed in DLS (P0–P3), which reflects a dynamic mixture of primary micelles, fragments, and aggregates (Figure 2a–d).

This shear-driven mechanism fundamentally alters the relationship between the chain conformation and reaction rate. While the constant disruption likely prevents the formation of long, stable *trains* (thereby reducing the coordination efficiency on a per-chain basis), the net complexation rate is paradoxically enhanced. This acceleration is driven by the vast increase in accessible PbBr_2_ dissociation sites on the newly created nanoparticle surfaces, leading to a surge in [PbBr_3_]^−^ production (Figure 5b_iii_). Most importantly, this energy-driven pathway largely negates the architectural constraints that govern static systems. As shown in Figures 3 and 4, all copolymer architectures, including the sterically hindered symmetric systems, exhibit rapid [PbBr_3_]^−^ formation under shear. This provides clear evidence that when sufficient mechanical energy is supplied, the complexation process is no longer dominated by subtle differences in copolymer architecture but by shear-induced particle fragmentation and collision frequency.

## Conclusions

This study demonstrates that polystyrene-*block*-poly­(2-vinylpyridine) (PS-*b*-P2VP) architecture and mixing dynamics critically govern PbBr_2_ complexation. Under static conditions, the process is kinetically limited by micellar diffusion and architecture. Asymmetric copolymers with long P2VP blocks (e.g., SV_13.5/47_) enhance PbBr_2_ dissociation by forming *train*-rich conformations, enabling direct pyridine-Pb^2+^ coordination in the ^sm^PRE_*x*,13.5/47_ solutions. Symmetric copolymers (SV_49/49_, SV_133/132_) with thick PS shells of symmetric copolymers act as steric barriers that kinetically arrest complexation in the ^sm^PRE_*x*,49/49_ and ^sm^PRE_*x*,133/132_ solutions. Dynamic mixing shifts the mechanism: shear forces fragment PbBr_2_ into nanoparticles, accelerating complexation independently of the copolymer symmetry. In a static system, micelle dimensions and chain conformations (*trains*, *loops*, *tails*) dictate coordination efficiency. Stirring promotes transient adsorption–desorption equilibria, stabilizing polydisperse emulsions through micelle–particle collisions. This establishes clear design principles for controlling lead halide precursor solutions. It reveals that while molecular design is critical in diffusion-limited systems, its constraints can be overcome by shear-dominated processing, an insight that is fundamental for the future scalable synthesis of BCP-templated precursor solutions.

## Supplementary Material



## Notes

TEM images, SAXS profiles, and comparison of micelle diameter calculated from SAXS fitting and DLS measurement (Figure S1); fitting parameter of SAXS profiles in Figure S1e (Table S1); number-weighted DLS profiles of ^sm^PRE_*x*,*n*/*m*_ precursor solutions as a function of time (Figure S2); time evolution of the mean number-weighted hydrodynamic diameter (*d*_H,num_) and PDI for each BCP system using static mixing (Figure S3); visual observation of precursor solutions over time for static mixing and dynamic mixing (Figure S4); absorbance spectra of neat PS_50.8k_ in TMB (Figure S5); TEM images of dried precursor solutions (^sm^PRE_*x*,*n*/*m*_) prepared with different PS-*b*-P2VP architectures (Figure S6); measured hydrodynamic diameters (*d*_H_) and derived diffusion properties for the neat block copolymer micelles in TMB (Table S2); time evolution of the mean number-weighted hydrodynamic diameter (*d*_H,num_) for each BCP system using dynamic mixing (Figure S7); TEM images of dried precursor solutions (^dm^PRE_*x*,*n*/*m*_) prepared with different PS-*b*-P2VP architectures (Figure S8); SAXS and WAXD profiles of ^dm^PRE_*x*,*n*/*m*_ solutions of various molecular weights of PS-*b*-P2VP (Figure S9); quantitative analysis of DLS spectra in Figure 2a–d (Figure S10); comparison of UV–vis absorbance from various neat and precursor solutions (Figure S11); Gaussian deconvolution of experimentally measured UV–vis spectra of ^sm^PRE_*x*,*n*/*m*_ for different PS-*b*-P2VP copolymers (Figure S12) (PDF)
