# Supplementary material for: Micelle-Mediated PbBr2 Complexation: Influence of Block Copolymer Architecture and Mixing Conditions
Source: Langmuir. 2025 Dec 3;41(49):33245–55. doi: 10.1021/acs.langmuir.5c04107 (PMC12713784; doi:10.1021/acs.langmuir.5c04107)
Supplement: Supplementary file 1 [file la5c04107_si_001.pdf]

Supporting Information

Micelle-Mediated $PbBr_2$ Complexation: Influence of Block Copolymer Architecture and Mixing Conditions

Belda Amelia Junisu, and Ya-Sen Sun*

Department of Chemical Engineering, National Cheng Kung University, Tainan 701, Taiwan

*corresponding author: Y. S. Sun (Email: yssun@gs.ncku.edu.tw)

Characterization of neat $SV_{n/m}$ block copolymer solutions

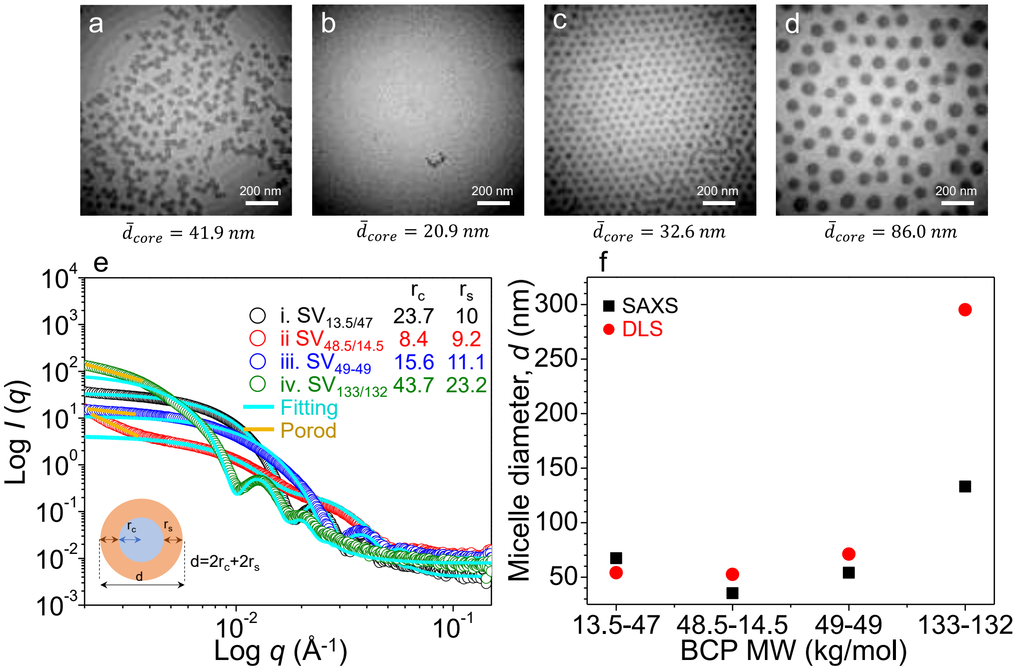

Figure S1. a-d) TEM images and e) SAXS profiles of neat micelles in 1 mg/mL solution of various PS-*b*-P2VP molecular weight; i. $SV_{13.5/47}$, ii. $SV_{48.5/14.5}$, iii. $SV_{49/49}$, and iv. $SV_{133/132}$. f) Comparison of micelle diameter calculated from the results of SAXS fitting and intensity-weighted DLS measurement.

Small-angle X-ray scattering (SAXS) was employed to characterize the micellar dimensions of neat PS-*b*-P2VP solutions in TMB. To interpret the scattering data, a core–shell sphere model[1] was applied. This model enables the extraction of important parameters such as the core radius (r_c), shell thickness (r_s), and the scattering length densities (SLDs) for the solvent,

P2VP core, and PS shell. At the dilute concentrations studied, the SAXS profiles reflected only form-factor scattering, with minimal contribution from structure factor effects, indicating minimal interparticle interactions. The fitting process involved modeling the scattering intensity, P(q), using the equation[1, 2]:

$$P(q) = \frac{Scale}{V} F^2(q) + background \quad (1)$$

where $P(q)$ represents the form factor which simplifies to unity under dilute conditions. The overall form factor contribution from a core–shell sphere is given by:

$$F(q) = \frac{3}{V_s}\left[V_c(\rho_c - \rho_s)\frac{\sin(qr_c) - qr_c\cos(qr_c)}{(qr_c)^3}\right] + \left[V_s(\rho_s - \rho_{sol})\frac{\sin(qr_{sp}) - qr_c\cos(qr_{sp})}{(qr_{sp})^3}\right] \quad (2)$$

where V_c and V_s denote the volumes of the core and shell, respectively, and ρ indicates the scattering length densities (SLDs) for each component (solvent 'subscript sol', core 'subscript c', and shell 'subscript s'). The radius of whole sphere denotes by r_{sp}. To account for the inherent size distribution of the micelles, the Schultz distribution function was incorporated into the fitting routine[3], allowing for an accurate assessment of polydispersity in the system. Moreover, the Porod model allows us to fit an intensity upturn with $I \sim q^{-\alpha}$ at low-q, given by:

$$Iq = Aq^{-\alpha} + B \quad (3)$$

where A and B are constants and α denotes the structural dimensionality.

Table S1. Fitting results of SAXS profiles in Figure S1e

**Fitting parameter**	**SV$_{13.5/47}$**	**SV$_{48.5/14.5}$**	**SV$_{49/49}$**	**SV$_{133/132}$**
r_c (nm); PDI$_c$ (nm)	23.68; 0.08	8.39; 0.43	15.63; 0.08	43.7; 0.09
r_s (nm)	10.00	9.19	11.14	23.2
$\rho_c - \rho_s$	0.5138	-1.401	0.5532	0.5992
$\rho_s - \rho_{sol}$	0.0437	0.8102	0.0519	0.0411

SAXS profiles of solutions with varying molecular weights (Figure S1e) displayed clear Guinier regimes at low-q, followed by oscillatory fringe patterns. These features are consistent with the formation of core-shell micelles displaying a polydisperse size distribution, which can be effectively modeled using a spherical core-shell model. A low-q upturn–absent in SV$_{13.5/47}$–indicated micelle clustering in for SV$_{48.5/14.5}$, SV$_{49/49}$, and SV$_{133/132}$. Power-law fitting of this low-q upturn yielded Porod exponents of -2.49, -0.31, and -0.83 for SV$_{48.5/14.5}$, SV$_{49/49}$, and

$SV_{133/132}$, respectively. The exponent for $SV_{48.5/14.5}$ suggests cluster structures with dimensionality intermediate between two and three, while $SV_{49/49}$ and $SV_{133/132}$ exhibited milder clustering. Notably, $SV_{48.5/14.5}$ displayed an additional scattering hump at q~0.02 $Å^{-1}$, absent in other copolymers. This feature likely reflects a less compact P2VP core[2] in $SV_{48.5/14.5}$, resulting in a pronounced scattering length density (SLD) contrast between the core and shell.

The apparent discrepancy between DLS and SAXS results can be attributed to differences in the fitted shell thicknesses and core radii, except for the $SV_{48.5/14.5}$ sample. In these systems, the shell thicknesses derived from SAXS are generally smaller than the corresponding core radii. Given that TMB is a good solvent for PS but poor solvent for P2VP, the PS shells are expected to be swollen, while the P2VP cores should remain in a collapsed, solvent-excluded state. When the PS and P2VP blocks have comparable molecular weights, the swollen PS corona is anticipated to be thicker than the compact P2VP core. However, the SAXS fitting model assumes a uniform SLD for the PS shell, which may not accurately capture the true structure. The PS corona likely exhibits an SLD gradient, with higher values near the PS/P2VP interface and lower values toward the periphery. In the outer corona, extended PS chains may have SLDs approaching that of TMB, making them nearly invisible to SAXS.

Analysis of the SAXS data for $SV_{13.5/47}$, $SV_{48.5/14.5}$, $SV_{49/49}$, and $SV_{133/132}$ revealed first-order scattering fringes position at q values of 0.023, 0.065, 0.037, and 0.013 $Å^{-1}$, respectively. Core–shell model fitting yielded the following core (r_c) and shell radii (r_s) as shown in Table S1. The corresponding total micelle diameters ($d_{SAXS}=2r_c+2r_s$) were 67.4 nm ($SV_{13.5/47}$), 35.2 nm ($SV_{48.5/14.5}$), 53.4 nm ($SV_{49/49}$), and 133.8 nm ($SV_{133/132}$). As shown in Figure S1f, SAXS-derived micelle diameters (d_{SAXS}) generally agreed well with intensity-weighted DLS measurements across the series, except for $SV_{133/132}$. The discrepancy observed for $SV_{133/132}$ is likely due to the higher polydispersity of micelles formed by this high molecular weight copolymer. It is noted that SAXS and DLS probe different physical quantities. SAXS measures the scattering intensity as a function of q and enables model-based fitting to extract structural parameters, including core size and corona thickness. In contrast, DLS determines hydrodynamic size through inversion of the time autocorrelation function, and the intensity-weighted signal scales with r^6, meaning that even trace amounts of larger aggregates can disproportionately influence the apparent distribution.

Figure S1a–d present TEM images of micelles formed by neat PS-*b*-P2VP in TMB. In these images, the dark regions correspond to the micelle cores, allowing for direct measurement

of core diameters. The average micelle diameters for $SV_{13.5/47}$, $SV_{48.5/14.5}$, $SV_{49/49}$, and $SV_{133/132}$ were determined using ImageJ software, yielding values of 41.9 nm, 20.9 nm, 32.6 nm, and 86.0 nm, respectively. These measurements are in good agreement with the core diameters obtained from SAXS fitting.

Distribution fits for the number-weighted DLS spectra of $^{sm}PRE_{x,n/m}$ solutions

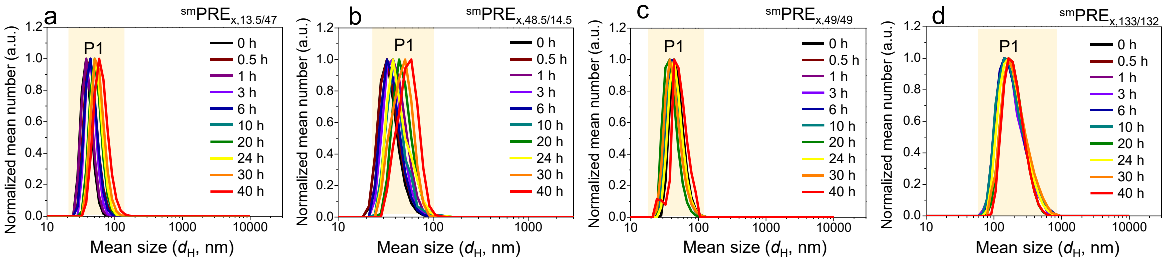

Figure S2. (a-d) Number-weighted DLS spectra of $^{sm}PRE_{x,n/m}$ precursor solutions measured over time (x: 0 – 40 h) for different copolymer molecular weights. The yellow highlighted regions in panels (a-d) indicate the primary micelle population (Peak 1, P1).

Figure S2a-d presents the number-weighted DLS spectra for the $^{sm}PRE_{x,n/m}$ solutions. In contrast to the bimodal feature observed in the intensity-weighted data (orange and red curves in Figure 1b), these number-weighted DLS spectra primarily show a single peak. To obtain a physically meaningful measure of the primary micelle size, all the spectra in Figure S2 were fitted with a Gaussian model. The resulting mean hydrodynamic diameters ($d_{H,num}$) are compiled and plotted in Figure S3. As shown, $d_{H,num}$ increased steadily over 40 hours for the $^{sm}PRE_{x,13.5/47}$ and $^{sm}PRE_{x,48.5/14.5}$ solutions, supporting our discussion that gradual complexation drives the slow growth of the primary micelles over time.

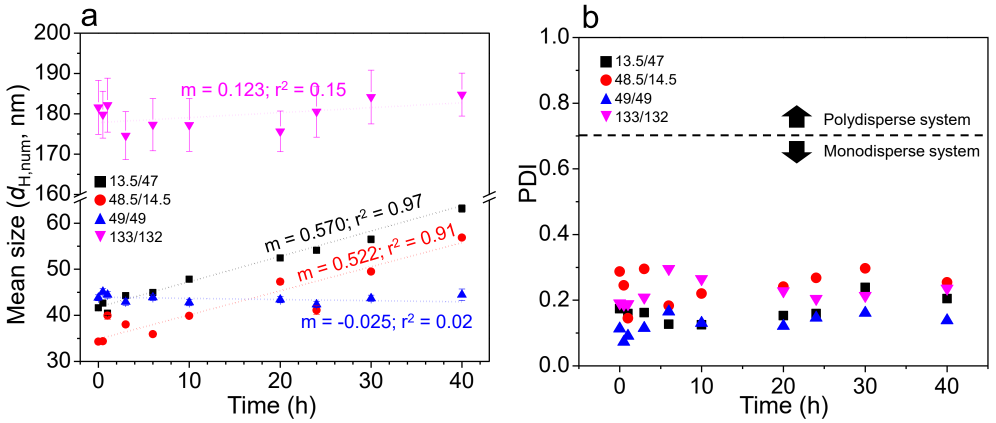

Figure S3. Time evolution of (a) the mean number-weighted hydrodynamic diameter ($d_{H,num}$) and (b) the PDI for the $^{sm}PRE_{x,n/m}$ solutions. The specific n/m ratios are 13.5/47 (black squares), 48.4/14.5 (red circles), 49/49 (blue triangles), and 133/132 (pink inverted triangles). In panel (a), $d_{H,num}$ values were obtained by Gaussian fitting of the number-weighted DLS spectra shown in Figure S2. In panel (b), PDI values were taken directly from the DLS analysis software.

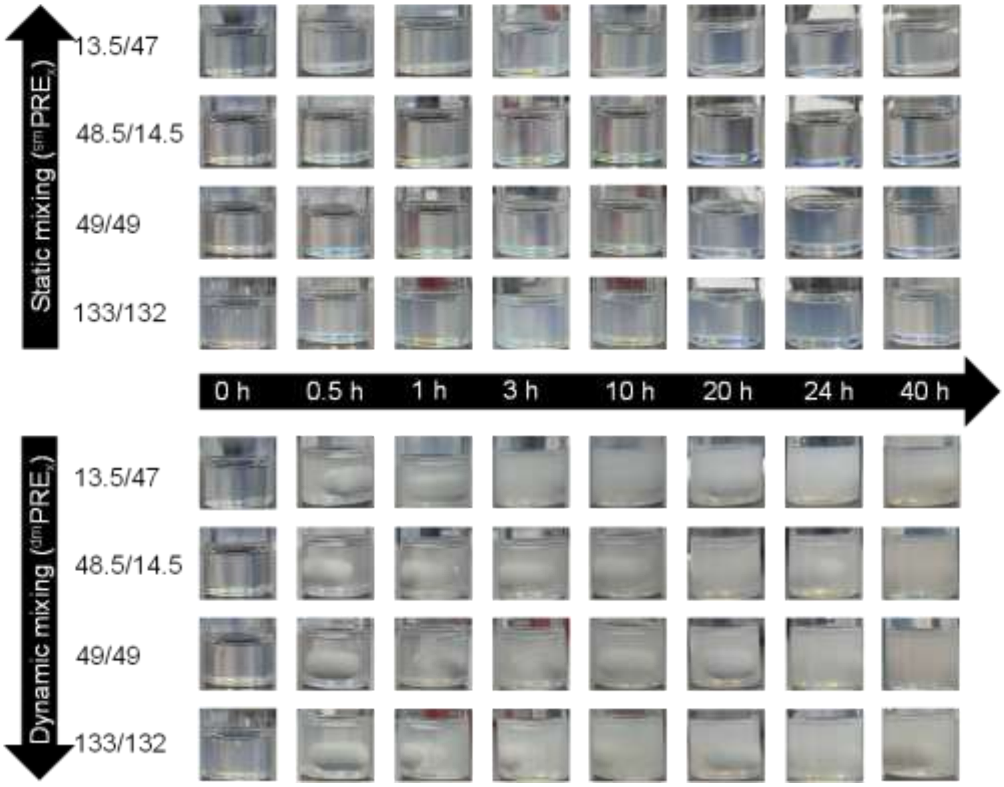

Figure S4. Visual observation of precursor solutions after static mixing ($^{sm}PRE_{x,n/m}$ upper panel) and dynamic mixing ($^{dm}PRE_{x,n/m}$, bottom panel) through storage time (for $^{sm}PRE_{x,n/m}$ solutions) or stirring time (for $^{dm}PRE_{x,n/m}$ solutions). The superscript “sm” and “dm” indicate static mixing and dynamic mixing, respectively.

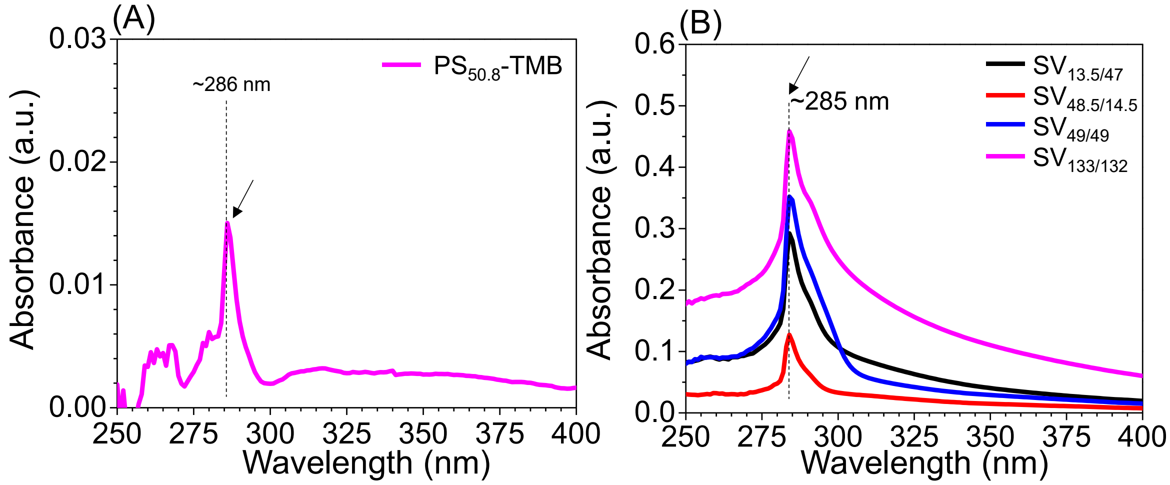

Figure S5. UV-vis absorbance spectra of (A) neat $PS_{50.8k}$ in TMB and (B) the neat $SV_{n/m}$ solutions.

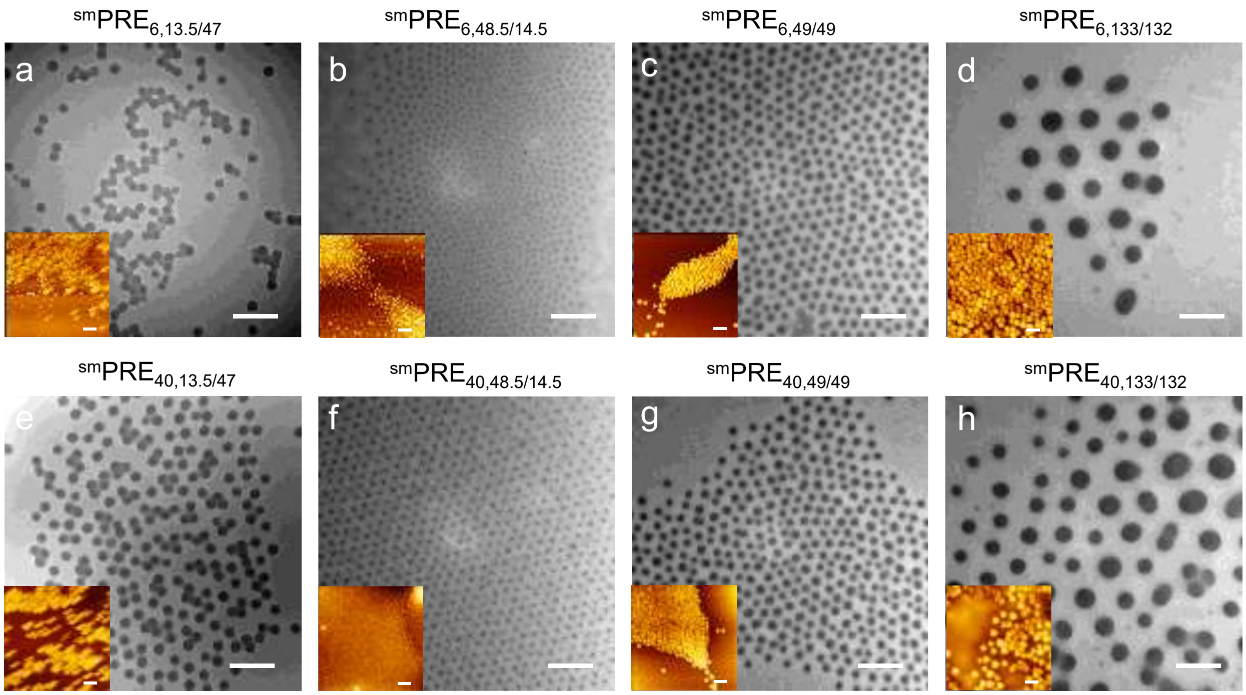

Figure S6. TEM images and AFM insets of dried precursor solutions ($^{sm}PRE_{x,n/m}$) prepared with different PS-*b*-P2VP architectures. The samples were stored under static mixing for (a-d) 6 h and (e-h) 40 h. The specific n/m for each column are: (a,e) 13.5/47, (b,f) 48.5/14.5, (c,g) 49/49, and (d,h) 133/132. All scale bars represent 200 nm.

Impact of micelle size on its transport properties

Table S2. Measured hydrodynamic diameters (d_H) and derived diffusion properties for the neat block copolymer micelles in TMB.

BCP	d_H (nm)	Calculated D (m^2/s)	Calculated t (s)
$SV_{13.5/47}$	56	1.20×10^{-11}	0.042
$SV_{48.5/14.5}$	52	1.29×10^{-11}	0.039
$SV_{49/49}$	71	9.45×10^{-12}	0.053
$SV_{133/132}$	296	2.27×10^{-12}	0.220

Note: Translational diffusion coefficients (D) were subsequently calculated using the Stokes-Einstein equation. The characteristic diffusion time (t) is the calculated time required for a micelle to diffuse a root-mean-square distance of 1 μm, based on the Einstein-Smoluchowski relation.

To quantitatively assess the impact of micelle size on its transport properties, we calculated the translational diffusion coefficient (D) and a characteristic diffusion time (t) for each block copolymer system. The translational diffusion coefficient of the micelles was calculated from the hydrodynamic diameter (d_H), as determined by Dynamic Light Scattering (DLS), using the Stokes-Einstein equation[4]:

$$D = \frac{k_B T}{3\pi\eta d_H} \quad (1)$$

where D is the diffusion coefficient, k_B is the Boltmann constant (1.38×10^{-23} J/K), T is the absolute temperature (assumed to be room temperature, 298 K), η is the dynamic viscosity of TMB (0.65×10^{-3} Pa·s at 298 K), and d_H is the hydrodynamic diameter of the micelle. The calculated diffusion coefficients for all neat micellar systems are summarized in Table S2.

To illustrate the kinetic consequences of these different diffusion coefficients, the characteristic time (t) required for a micelle to diffuse a root-mean-square distance $\langle x^2 \rangle$ was estimated using the Einstein-Smoluchowski relation[5]:

$$t = \frac{<x^2>}{2D} \quad (2)$$

We selected a relevant microscopic distance of 1 μm (1×10^{-6} m) to represent the length scale for a micelle approaching a $PbBr_2$ microparticle surface in the bulk solution. The results are shown in Table S2. This analysis demonstrates that while diffusion is rapid on the microsecond-to-millisecond timescale, there is still a clear and significant difference among the systems. The largest symmetric micelle requires more than five times longer to travel 1 μm compared to the

smallest asymmetric micelle, providing a quantitative basis for the observed differences in complexation kinetics under diffusion-controlled conditions.

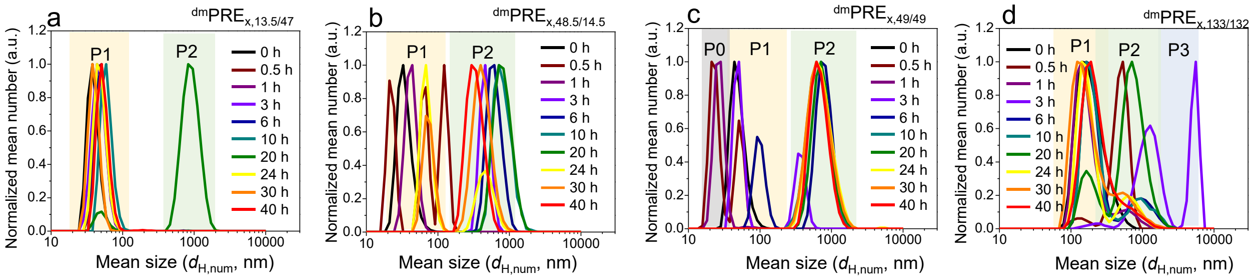

Figure S7. (a-d) Number-weighted DLS spectra of $^{dm}PRE_{x,n/m}$ precursor solutions measured over time (x: 0 – 40 h) for different copolymer molecular weights. Yellow, green, and blue highlighted areas in (a-d) correspond to peak 1 (P1), peak 2 (P2), and peak 3 (P3), respectively. Grey highlighted area denotes peak 0 (P0).

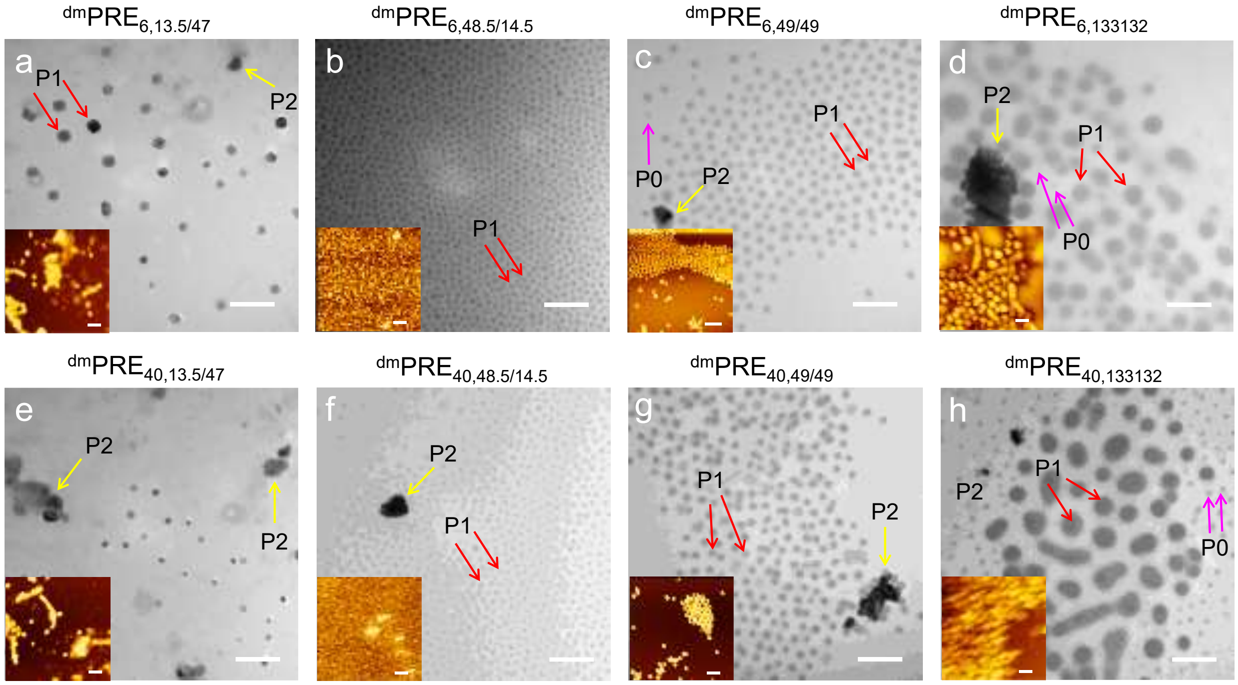

Figure S8. TEM images and AFM insets of dried precursor solutions ($^{dm}PRE_{x,n/m}$) prepared with different PS-*b*-P2VP architectures after (a-d) 6 h and (e-h) 40 h of stirring The specific n/m for each column are: (a,e) 13.5/47, (b,f) 48.5/14.5, (c,g) 49/49, and (d,h) 133/132. Pink, red, and yellow arrows correspond to the P0, P1, and P2 populations, respectively, as described in Figure 2. All scale bars represent 200 nm.

Characterization of $^{dm}PRE_{x,n/m}$ precursor solutions

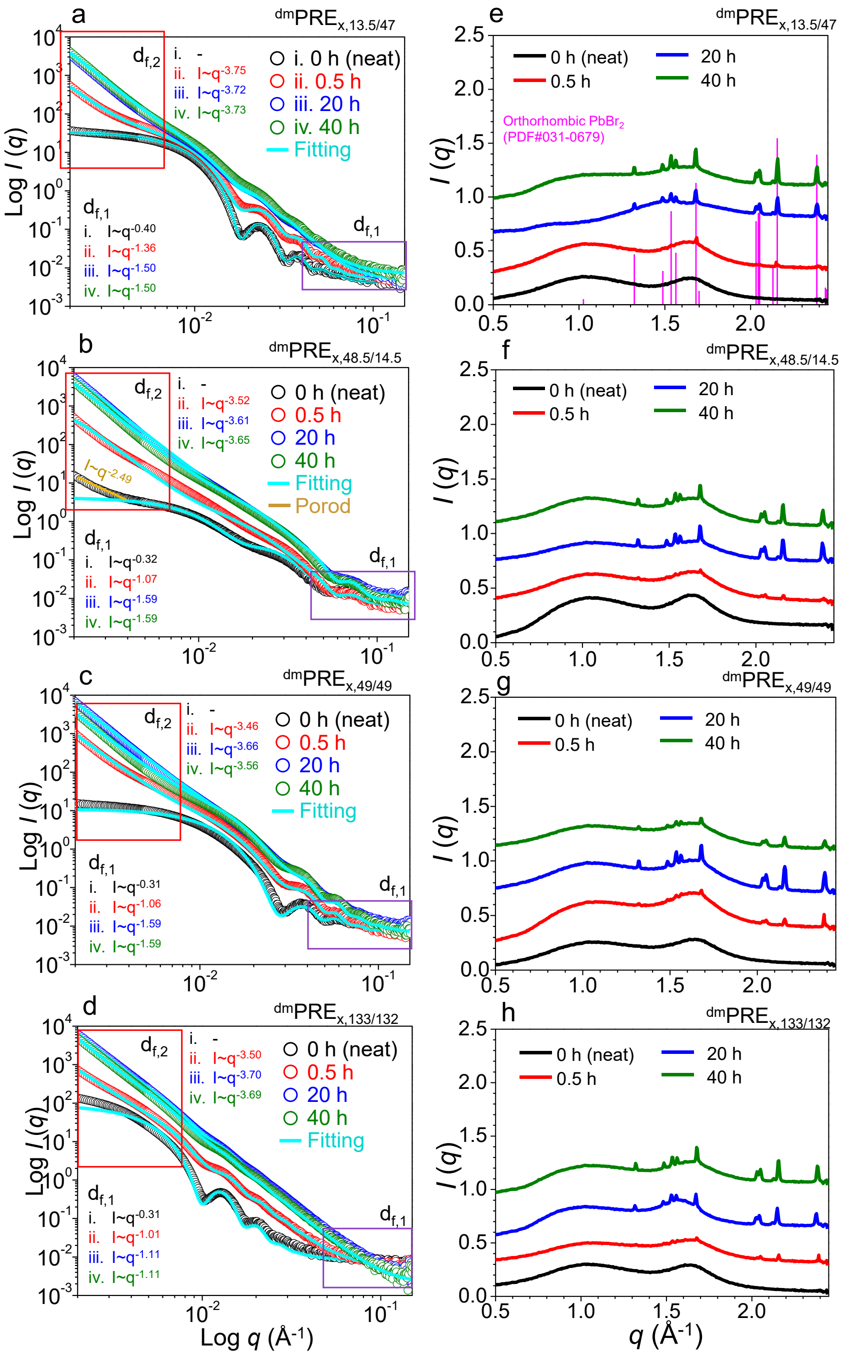

Figure S9. (a-d) SAXS and (e-h) WAXD profiles of $^{dm}PRE_{x,n/m}$ solutions prepared using different PS-*b*-P2VP architectures. The specific n/m for each row are: (a,e) 13.5/47, (b,f) 48.5/14.5, (c,g) 49/49, and (d,h) 133/132. Red and purple rectangles in SAXS profiles represent the low-*q* and high-*q* area which shows the evolution of fractal dimension $d_{f,2}$ and $d_{f,1}$ of $PbBr_2$/micelles dispersion, respectively. Vertical stick lines in (e) represent the diffraction peaks of orthorhombic $PbBr_2$ crystals (PDF#031-0679).

Two key features were observed in the scattering profiles in Figure S9a-d. First, the oscillatory fringes progressively diminish with increased stirring, suggesting core deformation due to surface adsorption. The presence of dispersed $PbBr_2$ microparticles likely contributes to enhanced diffuse surface scattering, which leads to the smearing of the the scattering fringes. Second, a pronounced upturn at low-*q* emerges, indicating the formation of compact microparticles that are stabilized by the surface absorption of PS-*b*-P2VP micelles. This low-q intensity upturn is attributed to increased surface scattering. Upon stirring, $PbBr_2$ microparticles are well-dispersed in TMB via a colloid-assisted microemulsion process. These microparticles are predominantly orthorhombic $PbBr_2$ crystals. Notably, the addition of PS-*b*-P2VP results only in $PbBr_2$ complexation, without triggering the phase transformations seen in the PS-*b*-PEO system[6].

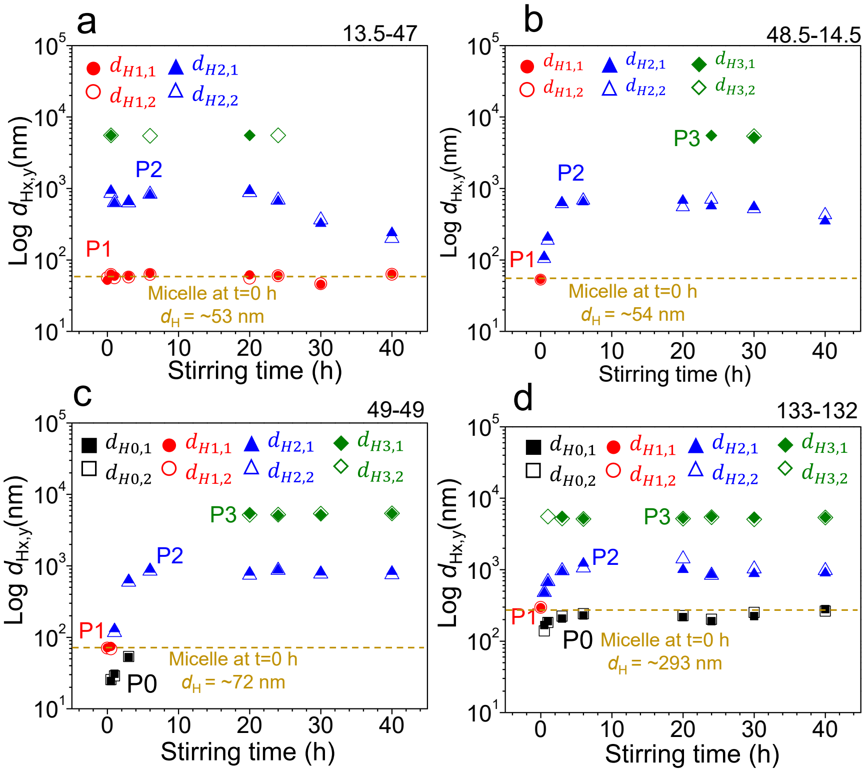

Figure S10. Quantitative analysis of DLS spectra in Figure 2a-d.

To quantitatively analyze the DLS spectra (Figure 2a-d), the hydrodynamic diameter (d_H) of each peak (P0, P1, P2, and P3) was tracked over time and plotted in Figure S10. The d_H for each PS-*b*-P2VP is represented as $d_{Hx,y}$, where 'x' denotes the peak number (P0-P3) and 'y' denotes the measurement sequence. Horizontal brown lines, indicating the d_H of neat micelles, serve as a visual

guide for the transition between P0 and P1. All PS-*b*-P2VPs displayed rapid P2 peak formation within the first 0.5 hours, while the P3 peak emergence varied more. The $^{dm}PRE_{x,49/49}$ and $^{dm}PRE_{x,133/132}$ solutions showed P0 peaks, suggesting potential micelle breakage under shear flow, a phenomenon not observed for the $^{dm}PRE_{x,13.5/47}$ and $^{dm}PRE_{x,48.5/14.5}$ solutions, likely due to their greater micelle stability. While DLS spectra interpretation for stirred precursor solutions is complex, the influence of molecular weight on diffusion and surface adsorption of micelles during $PbBr_2$ complexation appears less significant with stirring.

Optical properties on centrifuged precursor solutions

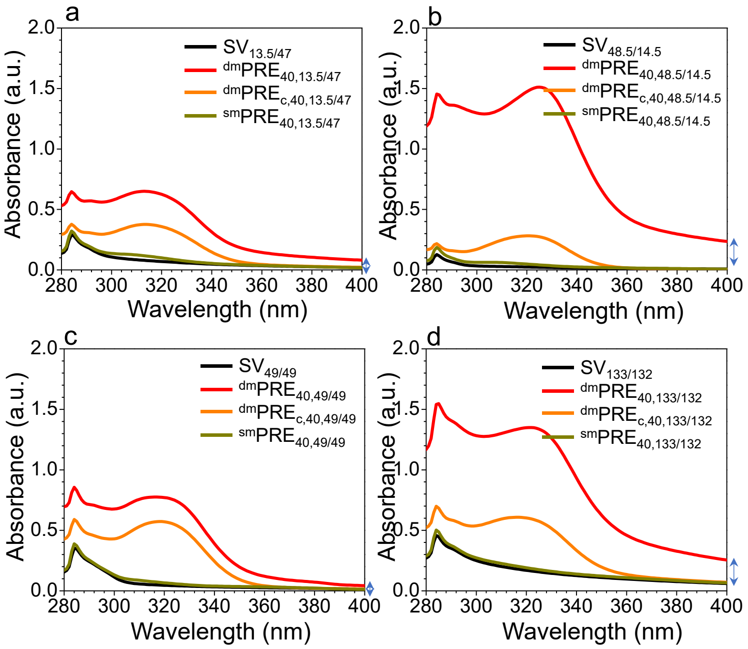

Figure S11. (a-d) UV-vis absorbance of solutions synthesized using different PS$_n$-*b*-P2VP$_m$ architectures. The specific n/m ratios are: a) 13.5/47, b) 48.5/14.5, c) 49/49, and d) 133/132.

Figure S11 compares the UV-vis spectra of neat PS-*b*-P2VP ($SV_{n/m}$), precursor solutions after 40 h of stirring ($^{dm}PRE_{40,n/m}$), centrifuged precursor solutions ($^{dm}PRE_{c,40,n/m}$), and precursor solutions prepared by static mixing ($^{sm}PRE_{40,n/m}$). The spectra of the uncentrifuged, stirred precursor solutions ($^{dm}PRE_{40,n/m}$) exhibit increased scattering due to dispersed $PbBr_2$ nanoparticles (blue arrows). This scattering is minimal in the centrifuged precursor solutions ($^{dm}PRE_{c,40,n/m}$; orange lines) and solutions prepared by static mixing ($^{sm}PRE_{40,n/m}$; lime green lines). Therefore, removing $PbBr_2$ through centrifugation not only remove the additional scattering but also remove

parts of the lead bromide complexes. Therefore, to isolate signals specific to coordination chemistry, all UV-vis spectra were baseline-corrected to account for scattering effects prior to analysis.

Deconvolution of UV-vis spectra for static mixing system

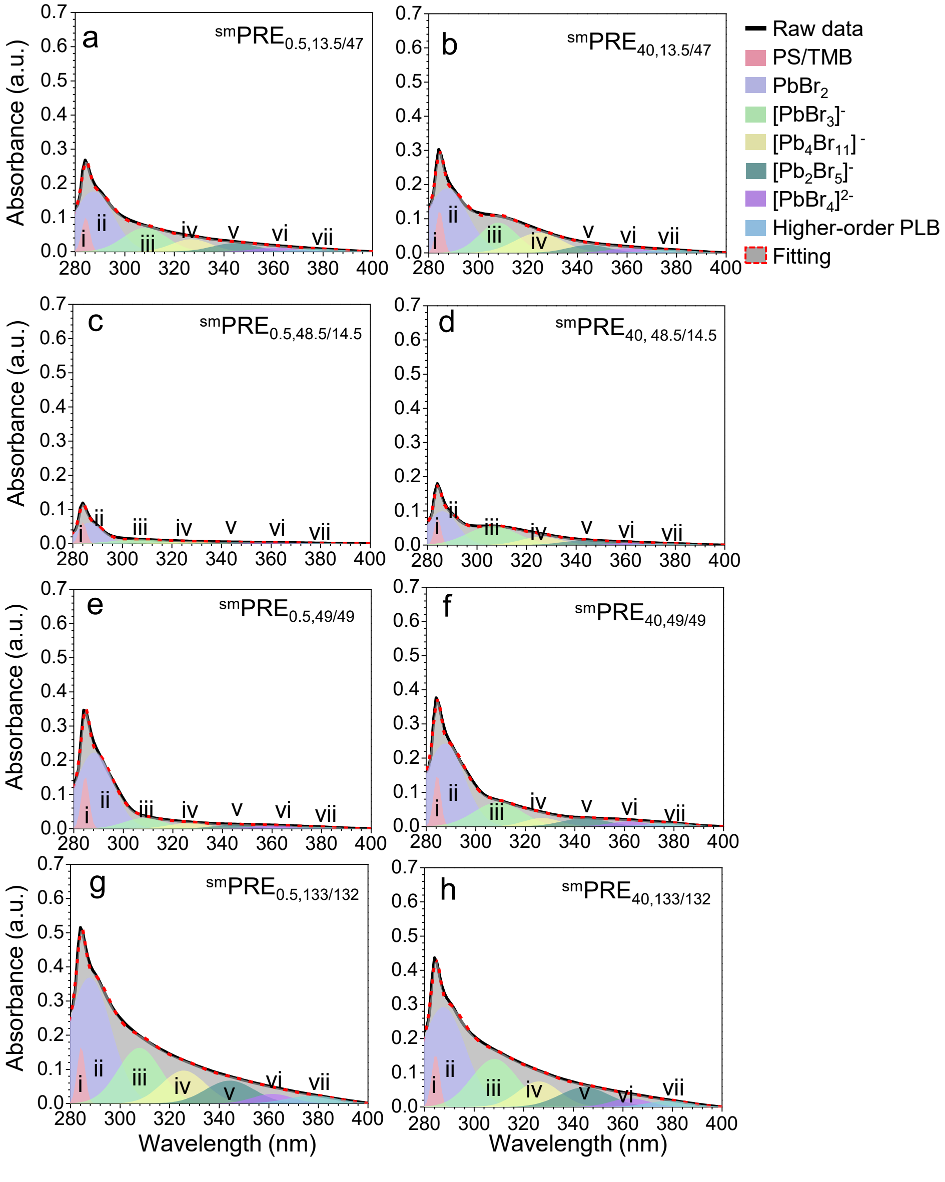

Figure S12. Experimentally measured UV-vis spectra of $^{sm}PRE_{x,n/m}$ for different PS-*b*-P2VP copolymers. Gaussian deconvolution of spectra after 0.5 h ($^{sm}PRE_{0.5,n/m}$) and 40 h ($^{sm}PRE_{40,n/m}$) of storage reveals seven distinct species-associated peaks, resolving contributions from (i) PS/TMB interactions (282–284 nm), (ii) micelle-dispersed $PbBr_2$ (289-292 nm), (iii) $[PbBr_3]^-$ (307–310 nm), (iv) $[Pb_4Br_{11}]^-$ (320-325 nm), (v) $[Pb_2Br_5]^-$ (343-345 nm), (vi) $[PbBr_4]^{2-}$ (357-360 nm), and (vii)

higher-order polynuclear lead bromoplumbate (PLB) complexes (370–380 nm). (superscript “sm”: static mixing)

References

1. Förster, S.; Timmann, A.; Konrad, M.; Schellbach, C.; Meyer, A.; Funari, S.; Mulvaney, P.; Knott, R., Scattering Curves of Ordered Mesoscopic Materials. *J. Phys. Chem. B* **2005,** *109*, 1347-1360.

2. Akiba, I.; Sakurai, K., Characterizing Block-Copolymer Micelles Used in Nanomedicines Via Solution Static Scattering Techniques. *J. Polym.* **2021,** *53*, 951-973.

3. Wang, S.-H.; Sun, Y.-S.; Chiang, A. S.-T.; Hung, H.-F.; Chen, M.-C.; Wood, K., Carboxylic Acid-Directed Clustering and Dispersion of Zro_2 Nanoparticles in Organic Solvents: A Study by Small-Angle X-Ray/Neutron Scattering and Nmr. *J. Phys. Chem. C* **2011,** *115*, 11941-11950.

4. Zmpitas, J.; Gross, J., Modified Stokes–Einstein Equation for Molecular Self-Diffusion Based on Entropy Scaling. *Ind. Eng. Chem. Res.* **2021,** *60*, 4453-4459.

5. Islam, M., Einstein–Smoluchowski Diffusion Equation: A Discussion. *Physica Scripta* **2004,** *70* (2-3), 120.

6. Chung, P.; Sun, Y.-S.; Zhao, B.-C.; Liu, C.-L., Template-Mediated Synthesis of Methylammonium Lead Bromide Quantum Nanodots with Tailored Optical Properties. *ACS Appl. Opt. Mater.* **2025**, *3*, 908-925.
